# Risk factors for infection with influenza A(H3N2) virus on a US university campus, October–November 2021

**DOI:** 10.1111/irv.13151

**Published:** 2023-05-25

**Authors:** Nathaniel M. Lewis, Miranda J. Delahoy, Kelsey M. Sumner, Adam S. Lauring, Emily E. Bendall, Lindsey Mortenson, Elizabeth Edwards, Aleksandra Stamper, Brendan Flannery, Emily T. Martin

**Affiliations:** ^1^ Influenza Division, National Center for Immunization and Respiratory Diseases CDC Atlanta Georgia USA; ^2^ Epidemic Intelligence Service CDC Atlanta Georgia USA; ^3^ University of Michigan School of Medicine Ann Arbor Michigan USA; ^4^ University of Michigan University Health Service Ann Arbor Michigan USA; ^5^ University of Michigan School of Public Health Ann Arbor Michigan USA

**Keywords:** congregate settings, influenza, respiratory infections, respiratory viruses, risk factors, student health

## Abstract

**Background:**

Knowledge of the specific dynamics of influenza introduction and spread in university settings is limited.

**Methods:**

Persons with acute respiratory illness symptoms received influenza testing by molecular assay during October 6–November 23, 2022. Viral sequencing and phylogenetic analysis were conducted on nasal swab samples from case‐patients. Case–control analysis of a voluntary survey of persons tested was used to identify factors associated with influenza; logistic regression was conducted to calculate odds ratios and 95% CIs. A subset of case‐patients tested during the first month of the outbreak was interviewed to identify sources of introduction and early spread.

**Results:**

Among 3268 persons tested, 788 (24.1%) tested positive for influenza; 744 (22.8%) were included in the survey analysis. All 380 sequenced specimens were influenza A (H3N2) virus clade 3C.2a1b.2a.2, suggesting rapid transmission. Influenza (OR [95% CI]) was associated with indoor congregate dining (1.43 [1.002–2.03]), attending large gatherings indoors (1.83 [1.26–2.66]) or outdoors (2.33 [1.64–3.31]), and varied by residence type (apartment with ≥1 roommate: 2.93 [1.21–7.11], residence hall room alone: 4.18 [1.31–13.31], or with roommate: 6.09 [2.46–15.06], or fraternity/sorority house: 15.13 [4.30–53.21], all compared with single‐dwelling apartment). Odds of influenza were lower among persons who left campus for ≥1 day during the week before their influenza test (0.49 [0.32–0.75]). Almost all early cases reported attending large events.

**Conclusions:**

Congregate living and activity settings on university campuses can lead to rapid spread of influenza following introduction. Isolating following a positive influenza test or administering antiviral medications to exposed persons may help mitigate outbreaks.

## 1 INTRODUCTION

As congregate settings with dense residential facilities and sustained social interactions, university campuses are common sites for outbreaks of influenza and influenza‐like illness (ILI).^1^, ^2^ In addition, campuses have high concentrations of young adults, who may be less likely to adopt behaviors that prevent or mitigate the spread of respiratory illness compared with other age groups.^1^, ^3^, ^4^, ^5^ Although both viral characteristics^6^, ^7^ and demographic characteristics^8^ may contribute to the likelihood of influenza, the social and environmental characteristics affecting the introduction and spread of influenza on university campuses are less understood.

Beginning in October 2021, a rapid increase in influenza A(H3N2) cases was reported by the University Health Service (UHS) at the University of Michigan in Ann Arbor, Michigan, USA, a university with more than 40 000 students, including approximately 11 000 who reside on campus. The unexpected outbreak represented some of the first substantial influenza activity reported during the COVID‐19 pandemic and followed a surge in COVID‐19 activity in Michigan.^9^ To characterize the influenza outbreak, CDC staff arrived on November 15, 2021, to further investigate risk factors associated with influenza infection, in collaboration with the university, Michigan Department of Health and Human Services, and local partners.^6^ Using a sample of students, staff, and university affiliates (e.g., visiting researchers and other employees) who received regular clinical testing for influenza during the outbreak, we retrospectively conducted viral sequencing and phylogenetic analysis of influenza‐positive specimens to assess virus evolution during the outbreak, an online survey to assess factors associated with influenza, and more detailed interviews with a subset of early case‐patients to better understand their exposures and the emergence of rapid transmission on campus.

## 2 METHODS

### 2.1 Study site and testing

University students, staff, and affiliates with symptoms of acute respiratory illness (ARI), including fever or chills, cough, shortness of breath or difficulty breathing, fatigue, muscle or body aches, headache, recent loss of taste or smell, sore throat, congestion or runny nose, nausea or vomiting, or diarrhea, were eligible to schedule an appointment for testing at the University Health Service (UHS), a clinic located centrally on campus. UHS staff collected a nasal swab among persons presenting for testing. Swabs were tested by a quad rapid multiplex molecular assay for influenza A, influenza B, SARS‐CoV‐2, and respiratory syncytial virus (RSV) at the University of Michigan.^2^ Cases were defined as persons receiving a positive test for influenza A at UHS during October 6–November 23, 2021 (no influenza B was detected); controls were defined as all persons testing negative for influenza during the same period, including those testing positive for SARS‐CoV‐2 or RSV. Among persons who received testing more than once during October 6–November 23, 2021, the first influenza A‐positive test result was used, or if the person never received an influenza A‐positive result, the first negative test result was used.

### 2.2 Viral sequencing and phylogenetic analysis

RNA was extracted from nasal swab samples (cycle threshold: ≤30) using the MagMAX viral pathogen nucleic acid isolation kit on a Kingfisher instrument. The influenza A virus genome was amplified by reverse transcription polymerase chain reaction (RT‐PCR) using universal influenza A primers^10^ and SuperScript IV master mix (Thermofisher). NEBNext companion kits were used for Nanopore library preparation. Samples were pooled (up to 96 samples per pool) and sequenced on an Oxford Nanopore GridION. For each sample, a consensus sequence was created using IRMA iterative refinement meta‐assembler. All samples were aligned to A/Darwin/9/2021 (clade 2a.2) using MAFFT.^11^ A phylogenetic tree was constructed using IQ‐TREE with a GTR substitution model, and a time tree was constructed using TreeTime. Molecular clock outliers were removed, and a revised time tree was constructed along with an estimate for the time to the most recent common ancestor. All data are currently available at the GISAID initiative (https://gisaid.org).

### 2.3 Survey and interview analysis

All persons tested during October 6–November 23, 2021, received an email prompt and a link to a survey with a combination of multiple‐choice and short‐answer questions asking about their demographic characteristics, residential information, symptoms during their illness, and other risk factors (e.g., travel and event attendance) potentially associated with influenza infection. A case–control approach was used to identify factors associated with infection. Logistic regression was used to calculate odds ratios (95% CIs) of factors potentially associated with influenza infection. Odds ratios with 95% CIs that did not span 1.0 were considered statistically significant and are denoted in tables by bold font. Qualitative telephone interviews were conducted with a convenience sample of 21 case‐patients who tested positive for influenza during October 6–November 2, 2021, the earliest phase of the outbreak (these case‐patients did not necessarily also respond to the survey). Interviews were 15–30 min in length and conducted during November 23–December 10, 2021, and targeted (1) initial sporadic cases reported in early October, (2) cases reported during the week after the October 18–19 fall break, and (3) cases reported during the week after the October 30–31 weekend when rapid transmission began. To identify factors contributing to the introduction and early circulation of influenza on campus, interviewers asked case‐patients semi‐structured questions about their residential situation, events attended prior to and during the first week of the outbreak, and how they thought they became infected with influenza.

All analyses were performed using SAS 9.4 (Cary, NC). All activities (i.e., testing, sequencing, surveys, and interviews) were determined to be non‐research public health surveillance by CDC and conducted consistent with applicable federal law and CDC policy (e.g., 45 C.F.R. part 46, 21 C.F.R. part 56; 42 U.S.C. Sect. 241(d); 5 U.S.C. Sect. 552a; 44 U.S.C. Sect. 3501 et seq).

## 3 RESULTS

**FIGURE 1:**
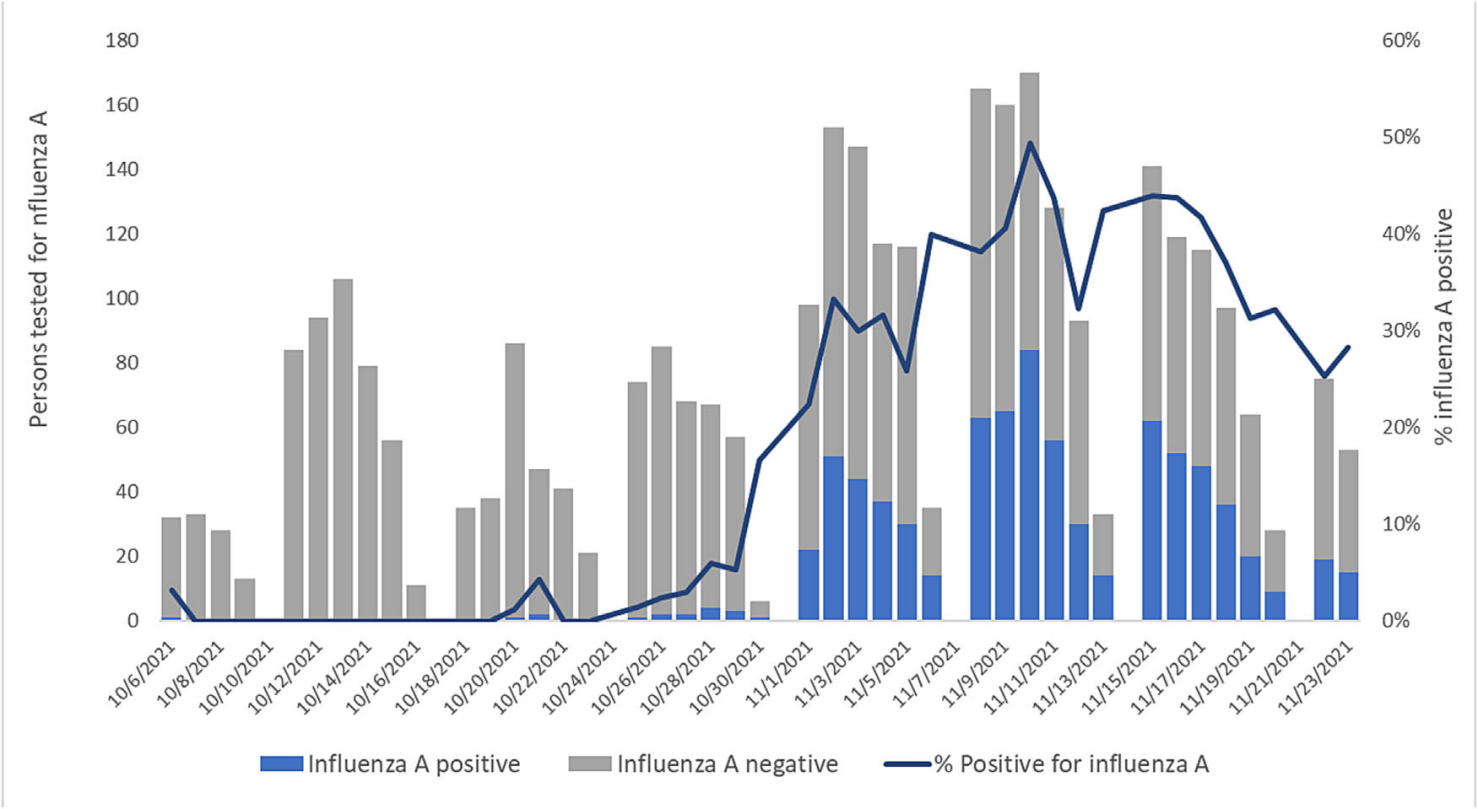
Number of persons tested for influenza A at University Health Services and percent influenza A positivity, by date of influenza test (*N* = 3268 tested)—University of Michigan, Ann Arbor, Michigan, USA, October 6–November 23, 2021. University Health Services does not conduct influenza A testing on Sundays. For persons tested more than once during October 6–November 23, 2021, the first influenza A positive test result was used, or if the individual never tested positive for influenza A, the first negative test was used.

During October 6–November 23, among 3268 persons tested, 788 (24.1%) tested positive for influenza A (Figure 1). In addition, of the 3268 persons tested, 143 (4.4%) tested positive for SARS‐CoV‐2 and 86 (2.6%) for RSV; five co‐infections of influenza and SARS‐CoV‐2 and six co‐infections of influenza and RSV were reported. No persons tested positive for influenza B. Among influenza case‐patients, the median age was 19.5 years (range: 17–31 years), and 53.3% were female.

**FIGURE 2:**
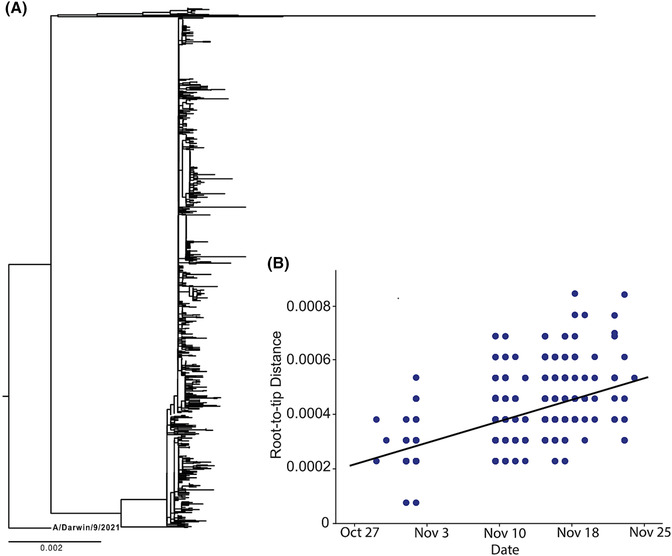
(A) Phylogenetic tree of human influenza whole genome sequences inferred using IQ‐TREE. Squares are collapsed outbreak samples with identical genotypes. (B) Regression from TreeTime showing the relationship between distance from the root and sampling time. The regression was used to calculate the molecular clock and the time to most recent common ancestor.

Whole genome viral sequences were obtained from 380 specimens with an earliest collection date of October 28, 2021; all viruses belonged to the A(H3N2) 2a.2 subgroup, which diversified recently from the influenza A(H3N2) subclade 3C.2a1b.2a viruses (i.e., full clade: 3C.2a1b.2a.2). Consistent with a rapidly spreading outbreak, there was little sequence diversity (Figure 2A). Using a whole genome phylogeny, we estimated the molecular clock to be 3.94 × 10.3 substitutions/site/year and estimated October 19, 2021, to be the time of the most recent common ancestor (Figure 2B).

Among 3268 persons tested, 744 (22.8%) responded to the survey only once and were included in the analysis. Of the 744 respondents, 207 (27.8%) tested positive for influenza A (case‐patients) and 537 (72.2%) tested negative (controls), of which 21/537 (3.9%) tested positive for SARS‐CoV‐2 and 19/537 (3.5%) tested positive for RSV. Patients with illness onset data (*n* = 659) received testing a median 2 days (interquartile range: 1–5 days) after onset. Among 193 case‐patients with data on influenza treatment, 89 (46.1%) reported being prescribed oseltamivir (i.e., Tamiflu). In addition, among the 637 who responded to survey questions regarding influenza prophylaxis, 45 (7.1%) reported being prescribed oseltamivir preventively.

The race/ethnicity of respondents was not associated with influenza infection (Table 1). Having university health insurance (versus private insurance) was associated with lower odds of influenza infection (OR: 0.42, 95% CI: 0.28–0.65); other types of insurance were not associated with odds of influenza. Odds of influenza was higher among those living with someone with ARI symptoms in the week prior to visiting the clinic (1.93 [1.34–2.78]). Likelihood of influenza varied by residence type and was higher among those residing in a shared living space, with elevated odds (relative to living alone in an apartment) observed among those living in an apartment with ≥1 roommate (2.93 [1.21–7.11]), a house other than a co‐op or fraternity/sorority (2.30 [1.30–4.08]), a single occupancy residence hall room (4.18 [1.31–13.31]), or in a residence hall room with ≥1 roommate (6.09 [2.46–15.06]). Odds were highest among those living in a fraternity or sorority house (15.13 [4.30–53.21]). Persons with residential contacts with ARI symptoms also had higher likelihood of influenza (1.93 [1.34–2.78]).

**TABLE 1:** Demographic and residential characteristics among persons tested for influenza at a US university, October–November 2021.

Characteristic	Influenza A positive (*N* = 207) *n*/*N* (%)^a^	Influenza A negative (*N* = 537) *n*/*N* (%)^a^	Odds ratio (95% confidence interval)^b^
Race/ethnicity			
Non‐Hispanic White	127/202 (62.9%)	362/525 (69.0%)	ref.
Hispanic/Latino	13/202 (6.4%)	22/525 (4.2%)	1.69 (0.82–3.44)
Asian, Non‐Hispanic	46/202 (22.8%)	98/525 (18.7%)	1.34 (0.89–2.00)
Black, Non‐Hispanic	3/202 (1.5%)	8/525 (1.5%)	**–**
Multiracial, Non‐Hispanic	11/202 (5.4%)	26/525 (5.0%)	1.21 (0.58–2.51)
Other Race, Non‐Hispanic	2/202 (1.0%)	9/525 (1.7%)	**–**
Student type			
In‐state	88/200 (44.0%)	249/527 (47.2%)	ref.
International	14/200 (7.0%)	28/527 (5.3%)	1.41 (0.71–2.81)
Other	1/200 (0.5%)	11/527 (2.1%)	**–**
Out‐of‐State	97/200 (48.5%)	239/527 (45.4%)	1.15 (0.82–1.61)
Type of health insurance			
Other private insurance	144/191 (75.4%)	301/506 (59.5%)	ref.
University health insurance	32/191 (16.8%)	158/506 (31.2%)	**0.42 (0.28–0.65)**
Medicaid	7/191 (3.7%)	24/506 (4.7%)	0.61 (0.26–1.45)
Other insurance	8/191 (4.2%)	23/506 (4.5%)	0.73 (0.32–1.67)
Residential situation			
Lives alone in an apartment	6/187 (3.2%)	53/468 (11.3%)	ref.
Lives alone in a residence hall room	9/187 (4.8%)	19/468 (4.1%)	**4.18 (1.31–13.31)**
Lives in a co‐op or a house with 10 or more people	4/187 (2.1%)	16/468 (3.4%)	**–**
Lives in a fraternity/sorority house	12/187 (6.4%)	7/468 (1.5%)	**15.13 (4.3–53.21)**
Lives in a residence hall room with ≥1 roommate	60/187 (32.1%)	87/468 (18.6%)	**6.09 (2.46–15.06)**
Lives in an apartment with ≥1 roommate	68/187 (36.4%)	205/468 (43.8%)	**2.93 (1.21–7.11)**
Lives in another type of house	28/187 (15.0%)	81/468 (17.3%)	**3.05 (1.18–7.87)**
Residential contact with ARI in the past week (ref = did not have residential contact with ARI in the past week)	72/175 (41.1%)	121/455 (26.6%)	**1.93 (1.34–2.78)**

^a^ The denominator represents the total survey respondents for each question.

^b^ Odds ratios are not presented when any cell count is <5.

Feeling very ill (by subjective self‐report) at the time of testing was associated with higher odds of influenza (2.33 [1.63–3.32]), as was suspecting influenza (3.92 [2.74–5.59]); suspecting COVID‐19 was associated with lower odds of influenza (0.55 [0.40–0.77]) (Table 2). The five most commonly reported symptoms among influenza case‐patients were runny nose and congestion (83.8%), fatigue (76.1%), headache (72.6%), muscle aches or joint aches (59.5%), and fever >100 degrees (57.4%); among those testing negative for influenza, the most commonly reported symptoms were runny nose or nasal congestion (71.2%), fatigue (43.8%), headache (43.3%), muscle aches or joint aches (23.8%), and difficulty breathing or shortness of breath (13.3%). Most symptoms reported were associated with an increase in the odds of influenza by 2–4 times; however, the effect estimate for fever was the strongest (9.42 [6.42–13.82]).

**TABLE 2:** Medical and care seeking risk factors among persons tested for influenza at a US university, October–November 2021.

Characteristic	Influenza A positive (*N* = 207) *n*/*N* (%)^a^	Influenza A negative (N = 537) *n*/*N* (%)^a^	Odds ratio (95% confidence interval)^b^
Reason for visiting clinic			
Felt very sick	143/197 (72.6%)	279/524 (53.2%)	**2.33 (1.63–3.32)**
Suspected COVID‐19	100/197 (50.8%)	341/524 (65.1%)	**0.55 (0.4–0.77)**
Suspected Influenza	141/197 (71.6%)	205/524 (39.1%)	**3.92 (2.74–5.59)**
Advised by friends/family to seek care	38/197 (19.3%)	82/524 (15.6%)	1.29 (0.84–1.97)
Always seeks care when sick	12/197 (6.1%)	20/524 (3.8%)	1.63 (0.78–3.41)
Has medical condition	7/197 (3.6%)	15/524 (2.9%)	1.25 (0.5–3.11)
Symptoms history			
Fever > 100 degrees	113/197 (57.4%)	65/520 (12.5%)	**9.42 (6.42–13.82)**
Runny nose or nasal congestion	165/197 (83.8%)	370/520 (71.2%)	**2.09 (1.37–3.19)**
Difficulty breathing or shortness of breath	50/197 (25.4%)	69/520 (13.3%)	**2.22 (1.48–3.35)**
Abdominal/stomach pain	19/197 (9.6%)	17/520 (3.3%)	**3.16 (1.61–6.76)**
Diarrhea	22/197 (11.2%)	29/520 (5.6%)	**2.13 (1.19–3.80)**
Nausea/vomiting	35/197 (17.8%)	30/520 (5.8%)	**3.53 (2.10–5.93)**
Headache	143/197 (72.6%)	225/520 (43.3%)	**3.47 (2.43–4.97)**
Muscle aches or joint aches	118/197 (59.9%)	124/520 (23.8%)	**4.77 (3.36–6.76)**
Ear pain or pressure	35/197 (17.8%)	95/520 (18.3%)	0.97 (0.63–1.48)
Fatigue	150/197 (76.1%)	228/520 (43.8%)	**4.09 (2.82–5.92)**

^a^ The denominator represents the total survey respondents for each question.

^b^ Odds ratios are not presented when any cell count is <5.

Several behaviors during the week prior to testing were also associated with influenza, as reported in the voluntary survey (Table 3). Off‐campus activities were associated with lower odds of influenza. These included leaving campus for ≥1 day to go to a friend's house, parents' house, or other unspecified location (0.49 [0.32–0.75]) and off‐campus travel to places where others were not wearing masks, including within Michigan (0.53 [0.33–0.85]) and outside of Michigan (0.27 [0.11–0.70]). However, dining indoors in a congregate setting (1.44 [1.00–2.03]), not wearing masks at indoor gatherings of ≥6 people (1.82 [1.27–2.62]) or at outdoor gatherings (2.33 [1.64–3.31]), and known contact with ≥1 person who had ARI (2.99 [2.00–4.46]) were all associated with increased likelihood of influenza.

**TABLE 3:** Behaviors among persons tested for influenza at a US university, October–November 2021.

Characteristic	Influenza A positive (*N* = 207) *n*/*N* (%)^a^	Influenza A negative (*N* = 537) *n*/*N* (%)^a^	Odds ratio (95% confidence interval)^b^
Non‐campus travel lasting ≥1 day during the week prior to visiting clinic^c^			
Went anywhere off‐campus	31/187 (16.5%)	138/476 (29.0%)	**0.49 (0.32–0.75)**
Maskless activities during the week prior to visiting clinic			
Dined indoors	117/188 (62.2%)	251/470 (53.4%)	**1.44 (1.02–2.03)**
Went to indoor gatherings of <6 people	124/188 (66.0%)	280/470 (59.6%)	1.31 (0.92–1.87)
Went to indoor gatherings of ≥6 people	133/188 (70.7%)	268/470 (57.0%)	**1.82 (1.27–2.62)**
Went to maskless outdoor gatherings	91/188 (48.4%)	135/470 (28.7%)	**2.33 (1.64–3.30)**
Went to a church, synagogue, mosque, or religious gathering	6/188 (3.2%)	18/470 (3.8%)	0.83 (0.32–2.12)
Traveled off‐campus, but within Michigan	25/188 (13.3%)	106/470 (22.6%)	**0.53 (0.33–0.85)**
Traveled outside of Michigan, but within the United States	5/188 (2.7%)	43/470 (9.1%)	**0.27 (0.11–0.70)**
Traveled outside of the United States	0/188 (0%)	5/470 (1.1%)	–
Contact in the week before influenza testing with persons who developed influenza‐like illness			
Contact with ≥1 person who developed ARI in the week before influenza test	114/158 (72.2%)	182/392 (46.4%)	**2.99 (2.00–4.46)**

^a^ The denominator represents the total survey respondents for each question.

^b^ Odds ratios are not presented when any cell count is <5.

^c^ Includes to friend's house, parents' house, or other unspecified location.

Among 21 early case‐patients interviewed with onset October 6–November 2, 2021, 19 (90%) reported how they thought they became sick (i.e., identified possible exposures or sources of infection), including four (21%) students who reported travel to Las Vegas with other students during October 15–20, 2021, and reported symptom onset during October 17–21. Additionally, four (21%) case‐patients reported attending a home football game on October 23 and symptom onset on October 25, and three (16%) reported attending an away football game at a nearby university on October 30 and had symptom onset during November 1–2. Five (26%) case‐patients reported potential exposures during Halloween weekend (October 30–31), mostly from large parties and at bars, and all had symptom onset during November 1–2.

## 4 DISCUSSION

Our findings highlight the potential for rapid transmission of influenza on university campuses. The timing of this outbreak was unusual given that substantial influenza activity during the last several pre‐pandemic seasons in Michigan generally had appeared around December and peaked around February.^12^ In addition, several COVID‐19 prevention initiatives updated in August 2021 were in place at the time of the outbreak, including required universal masking in most indoor spaces (with exceptions such as in dining halls while eating) during times of substantial or high community transmission of COVID‐19, and recommended masking at outdoor venues such as at sporting events (in addition to required COVID‐19 vaccination). Influenza vaccination was not required among students at the time of the outbreak and has not been made mandatory by the University other than for those working in its healthcare facilities.

Genomic sequencing suggested that a single ancestral clade of influenza virus was introduced and persisted with few mutations during the outbreak, supporting evidence of rapid on‐campus spread following introduction, despite respiratory infection precautions intended to reduce the spread of SARS‐CoV‐2. The similarity in antigenic clade observed for the duration of the outbreak further reinforces that most transmission likely occurred within the university community after introduction. Based on interviews, travel‐related events, such as an off‐campus trip, may have introduced influenza to campus, but student events (e.g., football games and Halloween parties) and residential environments (e.g., residence halls, fraternities, and sororities) likely contributed to early transmission on campus despite COVID‐19 prevention policies.

Once on‐campus transmission was present, the outbreak appeared to be driven largely by social and environmental factors. We observed that odds of influenza were highest in residential facilities most likely to have dense living quarters, which mirrors a previous influenza study showing increased odds of infection among those living in households of >3 persons (compared with ≤3 persons) and even greater odds among those living in households with >5 people.^13^ The highly increased odds of influenza diagnosis among those living in fraternity and sorority houses reinforces recent COVID‐19 network analysis studies showing that these residential facilities and the large events (e.g., parties, mixers, rush, and bid day) that members and residents attend are implicated in campus‐wide outbreaks.^14^ Maskless indoor and outdoor activities were similarly associated with increased odds of infection, suggesting that large outdoor activities (e.g., football games) as well as indoor ones may have been important sites of transmission, although these categories were not mutually exclusive, and many respondents may have engaged in both. Recent work on COVID‐19 has suggested that psychosocial characteristics typical of young adults, such as increased risk‐taking, social experience‐seeking, and less premeditation of consequences,^3^, ^14^ could have contributed to attending events or not masking during events or indoor interactions despite COVID‐19 recommendations.

Early case‐patients typically reported an interval of 2–3 days between presumed exposure and symptom onset, similar to that observed in previous influenza household transmission studies^15^ and thus potentially suggesting that exposure was sustained in terms of proximity to infected persons and amount of time spent with them.^16^ Rapid spread of influenza A(H3N2) on several university campuses in late 2021 raised concerns about a severe 2021–2022 influenza season in the community,^17^ potentially due to antigenic distance of the circulating virus from the vaccine or other factors.^3^, ^7^ However, the 2021–2022 influenza season in the United States was less severe than previous A(H3N2) seasons.^18^ This difference between the notably larger campus outbreaks and the subsequent, more moderate, community spread, however, suggests that university campuses have specific qualities that facilitate rapid spread (e.g., congregate living and activity settings) or that limit the implementation of the types of COVID‐19 preventive measures that may have been applied more consistently elsewhere. Although some associations may have differed slightly by excluding the small numbers of patients testing positive for SARS‐CoV‐2 and RSV from the control group, these patients were retained for representativeness of the population seeking testing at the time of the outbreak.

Several interventions may be effective in limiting the spread of influenza on university campuses. While several studies have demonstrated that vaccination remains the most important line of defense against influenza outbreaks, other interventions may be important during seasons in which vaccine effectiveness or uptake is lower. Past studies of influenza have suggested that students with ARI symptoms, while still undiagnosed, are still likely to attend classes, exams, social events, and trips,^4^ although they may avoid contact with others following a known influenza diagnosis.^3^ Providing isolation housing for students may reduce influenza transmission; however, this could be challenging in living settings where students have roommates and typically dine together. Interestingly, those who suspected they had influenza had higher odds of testing positive, suggesting that messaging could encourage persons who think they have been exposed to get tested and consider preventive measures to slow further transmission. Interviews with early case‐patients suggested, however, that many were unable to fully isolate due to needing to eat at indoor dining facilities. Access to centralized on‐campus testing, the desire to get tested during the COVID‐19 pandemic, and viral testing to detect both SARS‐CoV‐2 and influenza all likely facilitated the early detection of the influenza outbreak. While such tests could be useful for mitigating the spread of other respiratory illnesses (e.g., RSV) on university campuses, in this instance testing alone did not necessarily change the course of the outbreak, potentially suggesting a role for additional prevention measures.

Although more than half of respondents reported contact with someone with ARI during the week before their clinic visit and almost a third reported specifically having a residential contact with ARI, only 7% reported being prescribed oseltamivir after presenting for testing. Higher treatment rates (e.g., 20%–40%) have been recorded in studies of special populations, such as hospitalized patients, older adults in residential care settings, and children with chronic medical conditions.^19^, ^20^ Antiviral treatment among university students may represent an avenue for additional intervention given recommendations to consider using influenza antivirals for post‐exposure prophylaxis to prevent infection in communal settings (e.g., shelters, university dormitories, and prisons) and reduce strain on healthcare services in these settings during influenza outbreaks.^21^

Our findings are subject to at least five limitations. First, because transmission was already widespread when the investigation began, tour data collection tools could not identify specific potential exposures among most case patients. Second, as survey responses were collected 1–7 weeks following a positive test, the overall response rate was low. Relatedly, because respondents knew the results of their influenza tests at the time of responding to the survey, recall of exposures or risk behaviors may have been biased toward confirming a positive or negative test result. Third, as our survey only evaluated persons who chose to get tested, certain factors (e.g., frequency of mask wearing and adherence to hand hygiene) observed in this population may differ from the rate among all persons who had influenza during the outbreak at the university, including those who were not tested. Fourth, the list of factors evaluated for association with influenza diagnosis was not exhaustive and other factors, such as using shared versus private forms of transportation and visiting specific portions of campus during the week before symptom onset, could also be relevant. Finally, because questions about some risk behaviors (e.g., dining maskless indoors, attending indoor and outdoor events while maskless) were not location‐specific, this investigation did not identify specific locations of transmission, although genomic sequencing suggests that substantial transmission occurred rapidly within the university community, regardless of where students spent their time on campus.

## 5 CONCLUSIONS

Investigation of influenza outbreaks on university campuses can help identify risk factors for transmission, as well as the social and residential dynamics that can amplify odds of being infected. Risk factors for influenza inflection, including exposure to persons who are ill, and living in congregate settings, are similar to those for COVID‐19. In addition, introductions from one or two events can lead to rapid spread. Interventions, such as encouraging vaccination, testing, and quarantining following a positive influenza test, or use of preventive antiviral medication following exposure, could be helpful in mitigating outbreaks in environments such as college campuses.

## AUTHOR CONTRIBUTIONS

Nathaniel M. Lewis, Miranda J. Delahoy, and Kelsey M. Sumner led the field investigation and collection and analysis of survey and interview data. Adam S. Lauring and Lindsey Mortenson led the collection and analysis of case specimens. Emily Bendall, Elizabeth Edwards, and Aleksandra Stamper supported data collection and analysis. Emily T. Martin, Adam S. Lauring, Lindsey Mortenson, and Brendan Flannery provided supervision and resources. Emily T. Martin, Arnold S. Monto, and Adam S. Lauring obtained funding and provided supervision. Nathaniel M. Lewis drafted the manuscript. Nathaniel M. Lewis, Miranda J. Delahoy, Kelsey M. Sumner, Emily Bendall, Adam S. Lauring, and Emily T. Martin revised and finalized the manuscript.

## CONFLICT OF INTEREST STATEMENT

Emily T. Martin reports grants from NIH and Merck and payment for lectures from the Michigan Infectious Diseases Society, unrelated to the submitted work. Adam S. Lauring reports grants from the US CDC, National Institute of Allergy and Infectious Diseases, and Burroughs Wellcome Fund; consulting fees from Sanofi and Roche; and membership on the American Society of Virology governing council (unpaid), unrelated to the submitted work.

### PEER REVIEW

The peer review history for this article is available at https://www.webofscience.com/api/gateway/wos/peer-review/10.1111/irv.13151.

## Data Availability

The data that support the findings of this study are available from the corresponding author upon reasonable request. The data are not publicly available due to privacy or ethical restrictions.
